# Obesity and clinical outcomes in COVID-19 patients without comorbidities, a *post-hoc* analysis from ORCHID trial

**DOI:** 10.3389/fendo.2022.936976

**Published:** 2022-07-29

**Authors:** Peng Yu, Ziqi Tan, Zhangwang Li, Yi Xu, Jing Zhang, Panpan Xia, Xiaoyi Tang, Jianyong Ma, Minxuan Xu, Xiao Liu, Yunfeng Shen

**Affiliations:** ^1^ Department of Metabolism and Endocrinology, The Second Affiliated Hospital of Nanchang University, Nanchang, China; ^2^ Institute for the Study of Endocrinology and Metabolism in Jiangxi Province, Nanchang, China; ^3^ The Second Clinical Medical College of Nanchang University, Nanchang, China; ^4^ Department of Anesthesiology, The Second Affiliated Hospital of Nanchang University, Nanchang, China; ^5^ Department of Pharmacology and Systems Physiology, University of Cincinnati College of Medicine, Cincinnati, OH, United States

**Keywords:** obesity, COVID-19, comorbidities, death, discharge

## Abstract

**Objective:**

Large body of studies described individuals with obesity experiencing a worse prognosis in COVID-19. However, the effects of obesity on the prognosis of COVID-19 in patients without comorbidities have not been studied. Therefore, the current study aimed to provide evidence of the relationship between obesity and clinical outcomes in COVID-19 patients without comorbidities.

**Methods:**

A total of 116 hospitalized COVID-19 patients without comorbidities from the ORCHID study (Patients with COVID-19 from the Outcomes Related to COVID-19 Treated with Hydroxychloroquine among Inpatients with Symptomatic Disease) were included. Obesity is defined as a BMI of ≥30 kg/m^2^. A Cox regression analysis was used to estimate the hazard ratio (*HR*) for discharge and death after 28 days.

**Results:**

The percentage of obesity in COVID-19 patients without comorbidities was 54.3% (63/116). Discharge at 28 days occurred in 56/63 (84.2%) obese and 51/53 (92.2%) non-obese COVID-19 patients without comorbidities. Four (3.4%) COVID-19 patients without any comorbidities died within 28 days, among whom 2/63 (3.2%) were obese and 2/53 (3.8%) were non-obese. Multivariate Cox regression analyses showed that obesity was independently associated with a decreased rate of 28-day discharge (adjusted *HR*: 0.55, 95% *CI*: 0.35–0.83) but was not significantly associated with 28-day death (adjusted *HR*: 0.94, 95% *CI*: 0.18–7.06) in COVID-19 patients without any comorbidities.

**Conclusions:**

Obesity was independently linked to prolonged hospital length of stay in COVID-19 without any comorbidity. Larger prospective trials are required to assess the role of obesity in COVID-19 related deaths.

## Introduction

Obesity, traditionally defined as an excess of body fat causing prejudice to health usually with body mass index (BMI) of >30 kg/m^2^, is a serious global epidemic (1). As of late December 2021, there have been over 2 billion cases of COVID-19 and more than 5 million deaths reported worldwide. Both previous and our studies (2–4) showed a significant positive association between BMI and adverse outcomes in patients with COVID-19. Notably, in the COVID-9 pandemic, almost 94% of patients with COVID-19 co-exist with at least one comorbidity, and these comorbidities were higher in severe COVID-19 cases (5–7). These co-existing comorbidities, such as hypertension and diabetes, have been proven to be the strongest predictors of adverse outcomes in COVID-19 patients. Therefore, the effect of obesity on adverse outcomes in patients might be overestimated, even with full adjustment of those confounding factors in statistical processing. Despite the extensive work done on prognostic factors of COVID-19 and, considering that obesity often presents with other patient factors, limited data exist to investigate obesity in isolation of other co-morbidities in relation to COVID-19 prognosis. Given this background, we conducted this second analysis from the ORCHID (Patients with COVID-19 from the Outcomes Related to COVID-19 Treated with Hydroxychloroquine among Inpatients with Symptomatic Disease) trial, which is a multicenter, blinded, randomized clinical trial in the US that compared hydroxychloroquine with placebo during the clinical course of hospitalized patients with COVID-19, to examine the effect of obesity on prognosis in COVID-19 patients without comorbidities.

## Materials and methods

This research conforms to the Strengthening the Reporting of Observational Studies in Epidemiology (STROBE) statement (8).

### Data source

The detailed design and the main results of the ORCHID study have been previously reported (9). In brief, ORCHID is a multicenter, blinded, randomized clinical trial across 43 hospitals in the US that compared hydroxychloroquine with placebo during the clinical course of hospitalized patients with COVID-19. The trial included 479 hospitalized patients with COVID-19 confirmed with laboratory-confirmed SARS-CoV-2 positivity between 2 April 2020 and 19 June 2020. The Prevention and Early Treatment of Acute Lung Injury (PETAL) Clinical Trials Network Clinical Coordinating Center reviewed all the information to ensure the data quality. The ORCHID study was approved by the central institutional review board at the Vanderbilt University Medical Center. Informed consent for participation was obtained from all patients or their legally authorized representatives. The main results of the ORCHID study showed that COVID-19 patients treated with hydroxychloroquine, compared with placebo, did not significantly their improve clinical status on day 14 (9). Notably, the investigators of the RCTs were not involved in this study. This article was prepared using research materials obtained from the National Institutes of Heart, Lung, and Blood Institute.

We first excluded 35 patients with missing BMI (body mass index) values. Secondly, 349 patients with known pre-existing comorbidities at baseline were excluded (in detail described in **Supplemental Table S1**). Finally, 116 patients were included in the analysis.

### Study aim

Our present study provides evidence of the relationship between obesity and clinical outcomes in COVID-19 patients without comorbidities.

### Exposure

According to the recommendations of Body Mass Index (BMI) being classifications adopted by the World Health Organization (WHO) (1), obesity is defined as BMI ≥30 kg/m^2^. BMI was calculated as weight (kg) divided by height (m)^2^.

### Clinical follow-up

Patients were followed up for discharge and death following the trial randomization, and patient follow up included the use of in-hospital records and follow-up telephone interviews for discharged patients at 14 and 28 days after the trial randomization.

### Outcomes

The primary outcomes were a 28-day hospital discharge and a 28-day death. The detailed definitions of these outcomes were based on the previous descriptions (9).

### Statistical analysis

Baseline characteristics for continuous variables with normal distributions or nonnormal distributions were expressed as the means with standard deviations or medians with interquartile ranges, respectively. Categorical variables are expressed as frequencies and percentages. Comparisons between the groups were examined using unpaired Student’s t-tests (normal distribution) or the Wilcoxon–Mann–Whitney tests (nonnormal distribution) for continuous variance. Categorical variables with normal distribution or nonnormal distribution were compared using the χ^2^ tests or the Kruskal–Wallis test, respectively. Cox proportional hazards models were used to calculate the adjusted risk estimates (also known as hazard ratios [*HR*] and their confidence intervals [*CIs*]). Multivariate regression analysis was performed, and the confounding factor adjustment strategy was considered along with sample size, cases, and meaningful clinical confounding. Due to the limited sample size and cases, several principal confounding factors (age, sex, in-hospital use of corticosteroids, and SOFA score) were selected in this analysis.

## Results

### Baseline characteristics

One hundred and sixteen patients with COVID-19 without comorbidities were included in this study. **Table 1** shows the baseline characteristics of this cohort. Overall, the mean age of the cohort was 46.4 years, and 31% were females. The percentage of obese COVID-19 patients was 54.3% (63/116). There was no significant difference in age, sex, and other baseline characteristics stratified by obesity.

**Table 1:** Baseline characteristics of hospitalized patients with COVID-19 without comorbidities storified by obesity.

	Overall(n = 116)	Non-obesity(BMI <30 kg/m^2^n = 53)	Obesity(BMI ≥30 kg/m^2^n = 63)	*P-*value
**Demography**				
Age, years	46.36 (14.45)	46.81 (15.28)	45.98 (13.83)	0.76
Sex, male (%)	80 (69.0)	39 (73.6)	41 (65.1)	0.432
BMI, kg/m**^2^**	32.1 (7.1)	26.72 (2.0)	36 (6.3)	<0.001
Obesity I (%)	32 (27.6)	0 (0)	32 (50.1)	
**Home medication**				
Corticosteroids (%)	8 (6.9)	5 (9.4)	3 (4.8)	0.534
**Symptoms of acute respiratory infection**				
Cough (%)	70 (60.3)	31 (58.5)	39 (61.9)	0.854
Fever (%)	73 (62.9)	32 (60.4)	41 (65.1)	0.742
Shortness of breath (%)	81 (69.8)	36 (67.9)	45 (71.4)	0.836
Sore throat (%)	9 (7.8)	2 (3.8)	7 (11.1)	0.261
**Total SOFA score (mean (SD))**	2.94 (3.13)	2.70 (3.04)	3.14 (3.21)	0.448
**Measurements**				
Systolic blood pressure, mmHg	109 [99, 119]	109 [96, 117]	109.50 [105, 121]	0.189
Lowest SpO2, %	92 [90, 94]	92 [90, 95]	91.50 [90, 94]	0.246
Highest respiratory rate, breaths per minute	27.20 (8.33)	26.79 (9)	27.56 (7.76)	0.627
Hemoglobin, g/dl	13.45 [12.60, 14.72]	13.45 [12.62, 14.28]	13.55 [12.62, 14.97]	0.309
Sodium, mEq/L	136 [134, 138]	136 [134, 138]	136 [134.25, 138]	0.858
Potassium, mEq/L	3.80 [3.50, 4.10]	3.80 [3.50, 4.12]	3.80 [3.50, 4.10]	0.83
BUN, mg/dl	12 [9, 16]	12 [9, 17]	12 [9, 14]	0.57
AST, U/L	51.50 [38, 76.75]	50 [38, 80]	53 [37, 75]	0.809
ALT, U/L	47 [28, 72]	43.50 [33, 73.75]	48.50 [27.75, 63]	0.656
ALP, IU/L	76 [54, 90]	73 [55, 91.50]	76 [53, 89.75]	0.936
Bilateral opacities/infiltrates (%)	83 (74.8)	35 (68.6)	48 (80.0)	0.248
**Pre-medication up to randomization**				
Hydroxychloroquine (%)	3 (2.6)	2 (3.8)	1 (1.6)	0.879
Remdesivir (%)	7 (6.0)	3 (5.7)	4 (6.3)	1
Corticosteroids (%)	5 (4.3)	3 (5.7)	2 (3.2)	0.843
Tocilizumab (%)	3 (2.6)	3 (5.7)	0 (0.0)	0.185
Azithromycin (%)	37 (31.9)	15 (28.3)	22 (34.9)	0.574
**Medication between randomization and hospital discharge**				
Corticosteroids (%)	22 (19.0)	10 (18.9)	12 (19.0)	0.998
Tocilizumab (%)	11 (9.5)	3 (5.7)	8 (12.7)	0.332
Immunomodulating medication (%)	2 (1.7)	1 (1.9)	1 (1.6)	0.957

M(IQR) for nonnormally distributed data, M ± SD for normally distributed data, and n (%) for categoric variables.

SOFA, Sequential Organ Failure Assessment; BUN, blood urea nitrogen; ALT, alamine aminotransferase; AST, aspartate aminotransferase; ALP, alkaline phosphatase; BMI, body mass index.

### Association between obesity and death and hospital discharge

**Figure 1:**
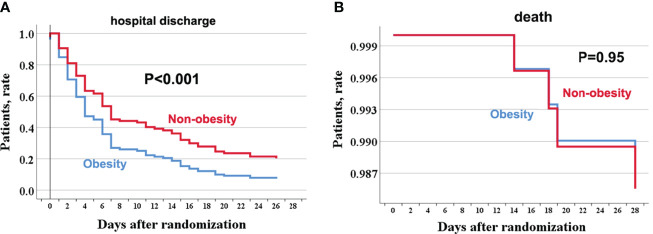
The age-adjusted incidence rate of hospital discharge and death through 28 days among obese and no-obese hospitalized patients with COVID-19 without comorbidities. hospitalization **(A)**; death **(B)**. For hospital discharge, all patients were followed up to discharge or 28 days after randomization in ORCHID. A patient was considered discharged from the hospital once discharged from the index hospitalization; rehospitalizations were not considered in this analysis. COVID-19, Corona Virus Disease 2019; ORCHID, Outcomes Related to COVID-19 Treated With Hydroxychloroquine Among Inpatients With Symptomatic Disease.

Discharge at 28 days occurred in 56/63 (88.9%) obese COVID-19 patients without comorbidities and in 51/53 (96.2%) non-obese COVID-19 patients without comorbidities. **Figure 1** shows the age-adjusted incidence of 28-day discharge across obesity and non-obesity groups, with a lower rate of 28-day discharge among obese patients (*p <*0.001). The association between obesity and outcomes by Cox regression is summarized in **Table 2**. Consistently, obesity was independently associated with a decreased rate of 28-day discharge after adjusting for age, sex, in-hospital use of corticosteroids, and SOFA score (adjusted *HR*: 0.55, *95% CI*: 0.35–0.83).

**Table 2:** Association between obesity and 28-day hospital discharge, and 28-day death in patients with COVID-19 without comorbidities.

	28-day discharge*	*P*-value	28-day death^#^	*P*-value
**Cases/N**	50/116		4/116	
**Crude HR** **(95% CI)**	0.72 (0.61–0.85)	**<0.001**	1.14 (0.16–8.07)	0.90
**Adjusted HR** **(95% CI)**	0.55 (0.35–0.83)	**<0.001**	0.94 (0.18–7.06)	0.95

*Adjusted for age, sex, in-hospital use of corticosteroid, and SOFA score.

#Adjusted for age.

SOFA, Sequential Organ Failure Assessment; HR, hazard ratio.

Four (3.4%) COVID-19 patients without any comorbidities died within 28 days, among whom 2/63 (3.2%) were obese, and 2/53 (3.8%) were non-obese. There was no significant difference in the age-adjusted 28-day death rate between obesity and non-obesity in COVID-19 patients without any comorbidities (*P* = 0.95). Further univariable and age-adjusted analyses showed similar results (adjusted *HR*: 0.94, *95% CI*: 0.18–7.06). Using sensitivity analysis by changing the 28-day death as in-hospital death or a composite of ECMO or in-hospital mortality, the results were similar to the primary analyses (data not shown).

## Discussion

To the best of our knowledge, this is the first study to explore the impact of obesity on COVID-19 patients without comorbidities. In this multicenter cohort study, we included 116 COVID-19 patients without any comorbidities from the US and found that obesity is associated with a decreased rate of 28-day discharge in COVID-19 patients without comorbidities as it had no significant effect on 28-day death.

Among patients with COVID-19, the prognosis could be worsened by co-existing disease burdens such as hypertension and cardiovascular diseases. Previous evidence showed that obese patients with COVID-19 have a worse prognosis, including respiratory and multiple organ failure and higher mortality (10) Nevertheless, most of the prior studies were based on patients with COVID-19 coexisting with comorbidities. Since obese patients often have these chronic conditions, this might be the most crucial reason for the increased risk of obesity in COVID-19 (11). Although the effect of comorbidities was adjusted, questions remain regarding the association and magnitude of the association between obesity and adverse outcomes in patients with COVID-19 without commodities. Our study excluded COVID-19 patients with any comorbidities and found that obese individuals are less likely to be discharged within 28 days than non-obese patients. However, our study did not find a significant association between obesity and the risk of 28-day death. Notably, we should explain these results with caution. First, we all know, the prevalence of comorbidities in children is much lower than that in adults. Multiple studies (12), according to a lower COVID-19 infection, severe cases, and cases in young adults or children, were observed. However, regarding death, the results were not consistent across children and young adults. In a small case-control study by Zhang et al. (13), obesity predisposes to higher death in young adults. In a large study from the UK, the relative risk of severe COVID-19 due to increasing BMI was found, particularly in people younger than 40 years old (14). A multi-center cohort study also found obesity, diabetes, and hypertension increase the risk of COVID-19-related mortality in young and middle-aged patients (15). In contrast, a cohort study of 795 children did not find increased mortality, although the length of hospital stays or severity was found (16). Secondly, since the mortality of this cohort was relatively low (3.4%), our study may be underpowered to detect such a difference; the *post hoc* power calculation showed that this study had a power of 4% to detect the difference observed (17). Finally, results from a UK national cohort found graded I obesity and severe obesity significantly increased the risk of mortality, rather than graded I obesity (30–34.9 kg/m^2^) (18). Notably, more than half of the patients in our cohort were grade I obese, which have diluted the statistical power. Therefore, based on current evidence, we cannot draw any final conclusion on the association of obesity with death in patients without comorbidities.

The potential mechanisms for the effect of obesity on COVID-19 length of hospital stay or severity have been extensively studied. Firstly, findings from the post mortem showed that pulmonary involvement was the dominant pathological feature. Increasing evidence indicates that obesity could result in altered lung physiology, including reduced lung volumes, abnormal ventilation, and perfusion distribution, decreased compliance, and respiratory muscle inefficiency (19–21). These changes subsequently induce ventilation–perfusion abnormalities and further reduce the ventilatory reserve, which makes the obese more prone to respiratory failure or even multiple organ failure after the infection of COVID-19 (22). Secondly, the weight gain and adipose tissue dysfunction in obesity could induce hyperinsulinemia/insulin resistance (23), metabolic tissue stress, resulting in chronic inflammation and further leading to the release of chemotactic mediators (24), which promote inflammatory leukocyte infiltration and secretion of pro-inflammatory cytokines (25), as well as complement system hyperactivation (26). These changes might ultimately develop a condition described as “cytokine storm,” the proposed mechanism that appears to drive severe COVID-19 infections (27). Finally, information on echocardiography in this study is not available. However, the hospitalized COVID-19 patients with obesity, even without comorbidities, may be associated with subtle myocardial dysfunction, which leads to worse clinical outcomes (28, 29).

### Strength and limitations

To the best of our knowledge, this is the first study exploring the association between obesity and clinical outcomes of COVID-19 in patients without comorbidities. However, the small sample size inevitably becomes the main limitation of our study. Studies with a larger sample size are needed to confirm our results. Secondly, our study is based on the US population, which limits the generation in other countries. Third, though being a typical measurement of obesity, BMI itself has various deficiencies, including its indirection of measuring body fat and of reflecting body fat distribution, as well as the changes in muscle mass (30). This might lead to a misinterpretation of the relationship between obesity and mortality. Other measurements, such as waist circumference, need to be further assessed. Thirdly, other confounding factors in COVID-19, such as race (31), physical activity (32), and fitness, are missing in our study.

## Conclusion

Obesity is linked to prolonged hospital length of stay in COVID-19 without comorbidities. The role of obesity on COVID-19 related deaths should be studied by larger prospective trials.

## Data availability statement

The original contributions presented in the study are included in the article/**supplementary material**. Further inquiries can be directed to the corresponding authors.

## Ethics statement

The studies involving human participants were reviewed and approved by ORCHID trial. The patients/participants provided their written informed consent to participate in this study.

## Author contributions

YS and XL were responsible for the entire project and revised the draft. PY, ZL, JZ, PX, and XT performed the data extraction, statistical analysis, interpreted the data, and drafted the first version of the manuscript. JM and MX revised the manuscript. All authors participated in the interpretation of the results, prepared the final version of the manuscript, and agreed to be accountable for all aspects of the work in ensuring that questions related to the accuracy or integrity of any part of the work are appropriately investigated and resolved.

## Funding

This work was supported in part by the Natural Science Foundation in Jiangxi Province grant (No. 202002BAB216022 to JZ, Nos. 20192ACBL21037 and 202004BCJL23049 to PY), the National Natural Science Foundation of China (Nos. 81760048 and 82160371 to JZ, Nos. 81760050 and 82100869 to PY). All fundings had no role in design, methods, subject recruitment, data collections, analysis, and preparation of the paper.

## Acknowledgments

We thank Outcomes Related to COVID-19 Treated with Hydroxychloroquine Among Inpatients With Symptomatic Disease (ORCHID) investigators for conducting this trial and making these data available. We acknowledge all healthcare workers involved in the fight against COVID-19 worldwide.

## Conflict of interest

The authors declare that the research was conducted in the absence of any commercial or financial relationships that could be construed as a potential conflict of interest.

## Publisher’s note

All claims expressed in this article are solely those of the authors and do not necessarily represent those of their affiliated organizations, or those of the publisher, the editors and the reviewers. Any product that may be evaluated in this article, or claim that may be made by its manufacturer, is not guaranteed or endorsed by the publisher.
