# Supplementary material for: Obesity and clinical outcomes in COVID-19 patients without comorbidities, a post-hoc analysis from ORCHID trial
Source: Front Endocrinol (Lausanne). 2022 Jul 29;13:936976. doi: 10.3389/fendo.2022.936976 (PMC9372447; doi:10.3389/fendo.2022.936976)
Supplement: Supplementary file 1 [file Table_1.docx]

**Supplement Table S1. Patients with known pre-existing chronic diseases at baseline**

| Chronic diseases | N=360 (%) |
| --- | --- |
| Diabetes mellitus | 164 (34.2%) |
| Cerebrovascular disease | 34 (7.1%) |
| Coronary artery disease | 42 (8.8%) |
| Myocardial infarction | 21 (4.4%) |
| Congestive heart failure | 35 (7.3%) |
| Peripheral vascular disease | 19 (4.0%) |
| Hypertension | 251 (52.4%) |
| COPD | 39 (8.1%) |
| Asthma | 48, 10.1% |
| Liver disease | 9 (1.9%) |
| Moderate to severe kidney disease | 42 (8.8%) |
| Dementia | 29 (6.1%) |
| Hemiplegia | 2 (0.4%) |
| Connective tissue disease | 19 (4.0%) |
| Peptic ulcer disease | 9 (1.9%) |
| Leukemia (AML, CML, ALL, multiple myeloma) | 3 (0.6%) |
| Malignant lymphoma | 3 (0.6%) |
| Solid tumor | 2 (0.4%) |

* Moderate to severe kidney disease was defined as Cr >3, ESRD, chart diagnosis of CKD stage 5 (eGFR <15 mL/min/1.73m²) not on dialysis

**Table S2: Baseline characteristics of included and excluded hospitalized patients with COVID-19**

|  | Excluded  (N=360) | Included  (N=116) | *P* value |
| --- | --- | --- | --- |
| Demography |  |  |  |
| Age, years | 60.19 (15.69) | 46.36 (14.45) | <0.001 |
| Sex, male (%) | 186 (51.8) | 80 (69.0) | 0.002 |
| BMI, kg/m2 | 33.05 (10.60) | 32.15 (7.05) | 0.395 |
| Obesity I (%) | 79 (21.9) | 32 (27.6) | 0.211 |
| Home medication |  |  |  |
| Corticosteroids (%) | 39 (10.8) | 8 (6.9) | 0.290 |
| Total SOFA score | 1.07 (0.90) | 0.98 (1.04) | 0.377 |
| Symptoms of acute respiratory infection |  |  |  |
| Cough (%) | 211 (58.6) | 70 (60.3) | 0.825 |
| Fever (%) | 197 (54.7) | 73 (62.9) | 0.149 |
| Shortness of breath (%) | 259 (71.9) | 81 (69.8) | 0.748 |
| Sore throat (%) | 25 (6.9) | 9 (7.8) | 0.929 |
| Measurements |  |  |  |
| Systolic blood pressure (median [IQR]) |  |  |  |
| Lowest SpO2, % | 92.00 [90.00, 95.00] | 92.00 [90.00, 94.00] | 0.411 |
| Highest respiratory rate, breaths per minute | 5.24 (1.71) | 4.95 (1.85) | 0.123 |
| Hemoglobin, g/dl | 12.50 [11.15, 14.00] | 13.45 [12.60, 14.72] | <0.001 |
| Sodium, mEq/L | 137.00[135.00, 139.00] | 137.00[135.00, 139.00] | 0.664 |
| Potassium, mEq/L | 4.00 [3.70, 4.40] | 3.90 [3.70, 4.20] | 0.088 |
| BUN, mg/dL | 17.00 [11.00, 29.00] | 12.00 [9.00, 16.00] | <0.001 |
| AST, U/L | 39.00 [28.00, 61.00] | 51.50 [38.00, 76.75] | <0.001 |
| ALT, U/L | 29.00 [18.00, 46.00] | 47.00 [28.00, 72.00] | <0.001 |
| ALP, IU/L | 75.00 [59.75, 97.00] | 76.00 [54.00, 90.00] | 0.466 |
| Bilateral opacities/infiltrates (%) | 207 (59.8) | 83 (74.8) | 0.006 |
| Pre-medication up to randomization |  |  |  |
| Hydroxychloroquine (%) | 1 (0.3) | 3 (2.6) | 0.074 |
| Remdesivir (%) | 17 (4.7) | 7 (6.0) | 0.751 |
| Corticosteroids (%) | 30 (8.3) | 5 (4.3) | 0.215 |
| Tocilizumab (%) | 2 (0.6) | 3 (2.6) | 0.183 |
| Azithromycin (%) | 111 (30.8) | 37 (31.9) | 0.927 |
| Medication between randomization and hospital discharge |  |  |  |
| Corticosteroids (%) | 65 (18.1) | 22 (20.0) | 0.272 |
| Tocilizumab (%) | 16 (4.4) | 11 (9.5) | 0.074 |
| Immunomodulating medication (%) | 2 (0.6) | 2 (1.7) | 0.539 |

**Notes:** M(IQR) for nonnormally distributed data, M ± SD for normally distributed data, and n (%) for categoric variables.

**Abbreviations:** SOFA, Sequential Organ Failure Assessment; BUN, blood urea nitrogen; ALT, alamine aminotransferase; AST, aspartate aminotransferase; ALP, alkaline phosphatase. BMI, body mass index
